# On the Importance of the Speed-Ability Trade-Off When Dealing With Not Reached Items

**DOI:** 10.3389/fpsyg.2018.00964

**Published:** 2018-06-13

**Authors:** Jesper Tijmstra, Maria Bolsinova

**Affiliations:** ^1^Methodology and Statisics, Tilburg University, Tilburg, Netherlands; ^2^University of Amsterdam, Amsterdam, Netherlands

**Keywords:** Item response theory (IRT), not reached items, item non-response, missing data, speed-accuracy trade-off, speed-ability trade-off, speeded power test, maximum performance test

## Abstract

In many applications of high- and low-stakes ability tests, a non-negligible amount of respondents may fail to reach the end of the test within the specified time limit. Since for respondents that ran out of time some item responses will be missing, this raises the question of how to best deal with these missing responses for the purpose of obtaining an optimal assessment of ability. Commonly, researchers consider three general solutions: ignore the missing responses, treat them as being incorrect, or treat the responses as missing but model the missingness mechanism. This paper approaches the issue of dealing with not reached items from a measurement perspective, and considers the question what the operationalization of ability should be in maximum performance tests that work with effective time limits. We argue that the target ability that the test attempts to measure is maximum performance when operating at the test-indicated speed, and that the test instructions should be taken to imply that respondents should operate at this target speed. The phenomenon of the speed-ability trade-off informs us that the ability that is measured by the test will depend on this target speed, as different speed levels will result in different levels of performance on the same set of items. Crucially, since respondents with not reached items worked at a speed level lower than this target speed, the level of ability that they have been able to display on the items that they did reach is higher than the level of ability that they would have displayed if they had worked at the target speed (i.e., higher than their level on the target ability). Thus, statistical methods that attempt to obtain unbiased estimates of the *ability as displayed on the items that were reached* will result in biased estimates of the *target ability*. The practical implications are studied in a simulation study where different methods of dealing with not reached items are contrasted, which shows that current methods result in biased estimates of target ability when a speed-ability trade-off is present. The paper concludes with a discussion of ways in which the issue can be resolved.

## 1. Introduction

In most applications of high- and low-stakes tests for ability measurement some form of time limit is imposed, either with the express intent to force respondents to work at a certain speed on the test, or simply because of practical considerations. Regardless of the reason why such time limits are imposed and whether they are imposed at the test level or at the subtest level, they impose a constraint on the speed at which the respondents can work while taking the test, as there is only a limited amount of time available to them to solve the items.

In a maximum performance test, test instructions will usually instruct the respondent to complete as many items correctly within the given time limit as possible. In line with this, most respondents will attempt to complete all the items on the test within the given time limit, to make use of their skills to manage the available time, and to adjust their speed accordingly. However, if the time limit is very restrictive, or if the instructions are not clear or not carefully read, a notable portion of the respondents may fail to complete the test within the time limit. Even with less restrictive time limits and clear instructions, not all respondents will succeed in managing their time to ensure a timely completion of the test. When respondents do not reach the end of the test on time, their responses to the not reached items are missing.

In large-scale educational assessment the amount of missing data due to not reached item is often not negligible (Pohl et al., 2014). For example, in the reading test of the German National Education Panel Study the percentage of not reached items was 10.5% for fifth-graders and 4.6% for nine-graders (Köhler et al., 2015). In PISA 2006 and 2009 the total percentages of missing responses due to not reached item were 4% (OECD, 2009, 2012). In TIMSS 2003, 3.73% of the items in Grade 8 and 5.96 % of the items in Grade 4 were not reached (Mullis et al., 2004). Numbers such as these suggest that answering the question how to statistically deal with missingness due to not reached items can be of great importance for the validity of measurement, as it affects how we deal with an often sizeable portion of the data matrix on which our statistical inferences rely.

It may be noted that missingness due to not reached items is not the only type of missing responses in maximum performance tests, as respondents may also have omitted responses (i.e., decided not to provide an answer to an item that was reached). There are at least two important differences that make distinguishing between these two types of missingness important, both for the conceptual discussion of the missingness phenomenon and its statistical treatment. First, omitted responses may occur anywhere in the test, while not reached items occur at the end of the test. This entails that the missingness mechanism differs: While missingness due to omission could in principle be thought to occur independently on different items, missingness due to not reached entails a strong dependence, since after the first item that was not reached, all the subsequent items will also have missing responses. Second, while missingness due to not having reached an item can be thought to be independent of item content and of the response that would have been obtained had the item been reached, this assumption is not plausible for omitted items (Mislevy and Wu, 1996). That is, an omitted item is an item where the respondent has had the opportunity to consider the item, but decided not to provide a response. On the other hand, the fact that an item was not answered by a respondent because they did not reach that part of the test means that such missingness is not related to the specific content of the item, even if it does tell us something about the speed at which the respondent worked (which may be related to their overall ability). Because of these two crucial differences between the two types of missingness, it is important to separate the discussion and treatment of missing responses due to not reached from those due to omission, and statistical procedures for dealing with them will have to rely on different assumptions^1^. In this paper the focus will be on missingness due to not reached items.

A variety of methods for addressing missing responses due to not reached items have been proposed in the literature. Very generally, one can distinguish three main ways of dealing with these not reached items for the purpose of ability measurement: (1) ignoring the missing responses, (2) replacing the missing values with a constant, or (3) taking the missing responses into account in the measurement model for ability.

The first approach to dealing with missingness due to not reached items is to simply ignore all these missing responses for the estimation of ability, and treat these items as not administered. This option can be defended by arguing that we simply did not get the chance to observe if the respondent would have solved the item if they would have reached it. Ignoring these missing responses is relatively straightforward if the data are analyzed using item response theory (IRT; see e.g., Hambleton and Swaminathan, 1985; van der Linden and Hambleton, 1997), as IRT models do not require observations on all the items on the test to be able to estimate the ability of a respondent (Lord, 1974). This option means that ability is estimated based only on the responses that were observed, with not reached items not having any impact on the estimated ability of the respondent. Missing responses due to not reached items are treated in this way for example in the National Assessment of Educational Progress (Allen et al., 2001).

A second and commonly considered option is to give the lowest amount of credit for not reached items. The idea behind this approach is that respondents were tasked to show maximum performance on each of the items on the test, and that they failed to show any performance on these not reached items. Hence, they should be awarded credit on these items in line with this (absence of) performance. A commonly used option is to impute a 0-value as the item score on the not reached items. This is in line with the explicit or implicit expectations that respondents will commonly have of how the test is scored if no information to the contrary has been provided in the test instructions. That is, unless informed otherwise, most respondents will assume that they get credit for each item answered correctly, and no credit for items for which no correct (or no partially correct) answer is given, which includes not reached items (Ludlow and O'Leary, 1999). Often, the item parameters are estimated given the observed data only while when estimating the person parameters zeros are imputed for the missing responses, as is done in large-scale educational surveys like PISA, TIMMS and PIRLS (see e.g., Martin et al., 2007; Olson et al., 2008; OECD, 2012).

One could defend this imputing of zero scores on not reached items by pointing out that the respondents essentially displayed zero performance on those items, and hence should get the lowest amount of credit. However, for items on which guessing results in a non-zero probability of success, imputing zero scores can be considered to be overly punitative, since the expected item score for a respondent with infinitely low ability would still be taken to be larger than zero. As suggested by Lord (1974), in such cases it may be more defendable to impute item scores that are in line with this probability of guessing the correct answer. For multiple choice items, one could pick this imputed score value a priori based on the number of response options (Lord, 1983).

The third option is to in some way take the missing responses into account in the estimation of ability. In line with the first approach, this means that one acknowledges that the respondent did not show any performance on the item, but the assumption that missing responses can simply be ignored is dropped. That is, the fact that an item was not reached can now be taken to be relevant information for the estimation of ability. Since the assumption of ignorability is dropped, the third option extends the approach of the first option by allowing the missingness to play a role in the estimation model.

Advanced psychometric approaches have been mainly focused on dealing with the fact that missing data due to items not being reached are not random (Mislevy and Wu, 1996) but rather are often related to ability (Rose et al., 2010; Pohl et al., 2014) and therefore non-ignorable. The idea is to model the respondents' tendency to have missing values and its relationship to the measured ability, which can be done either by modeling the latent missingness propensity (Glas and Pimentel, 2008; Debeer et al., 2017) or by using the number of not reached items in the model either as a continuous covariate (with linear and possible non-linear effects) in a latent regression or as a grouping variable in a multi-group IRT model (Rose et al., 2010, 2017; Rose, 2013).

The performance of these three different approaches has been investigated in a variety of simulation studies (Huisman, 2000; Pohl et al., 2014; Debeer et al., 2017). The general outcome of these studies has been that advanced psychometric methods in line with the third approach to dealing with not reached items result in relatively unbiased estimates of person parameters in the IRT model if the goal is to recover *the ability level displayed by the respondent on the items that were reached*, compared to the other two approaches. However, such simulation studies leave an important question unanswered, namely: How do the ability estimates that are obtained for respondents with not reached items relate to their level *on the ability that the test intended to measure*? That is, because respondents with not reached items worked at a speed level that was lower than the minimum speed level that the test instructed them to use, it is not a given that the ability level they displayed on the reached items fully matches what the test intended to measure.

To be able to determine to what extent the ability as estimated by the different procedures matches the ability of interest, we first need to answer a crucial question of measurement: What exactly is the ability of interest that the test intends to measure? This question needs to be addressed first before one can determine whether applying a certain statistical methodology will be helpful or hurtful for the measurement of that ability. That is, each of the three options will have a different impact on the ability estimates obtained for respondents with not reached items, and depending on our conclusion of what exactly the ability parameter is that we would ideally like to recover different methods may end up being preferred. Thus, the question what exactly we should take to be the ability of interest on a maximum performance test that works with time limits is of crucial importance for determining how we should deal with not reached items. This question and its implications for the question of how to best deal with not reached items on maximum performance tests are the topic of the current paper.

The structure of the paper is as follows. Section 2 deals with the speed-ability trade-off phenomenon, where its implications for the measurement of ability in maximum performance tests with time limits are discussed. Section 3 presents a simulation study in which the recovery of the desired ability parameter using statistical methods in line with the three different approaches to dealing with not reached items are considered under a variety of relevant conditions. The paper concludes with a discussion.

## 2. The influence of the speed-ability trade-off on ability measurement

### 2.1. The speed-ability trade-off

In IRT, a respondent's ability is taken to be a person property that together with relevant item properties (e.g., item difficulty and discrimination) explains observed differences between respondents in their item scores. Ability as it is estimated in IRT models thus captures the “effective ability” or “realized ability” of a respondent as it is displayed by the performance on the test, which need not be exactly the ability level that the test intended to measure (i.e., the “target ability”). For example, if a respondent has low motivation, their displayed effective ability may be lower than their level on the target ability of the test, which presumably concerns the ability that would be displayed if the respondent had aimed at maximum performance in the test given the time limit. However, when estimating the ability parameter of this respondent, the model will rely on the responses that were provided by this unmotivated respondent, implicitly assuming that they reflect their maximum performance on the test just as this is assumed for all the other respondents. Thus, the ability parameter in IRT models corresponds to a respondent's level of effective ability as displayed on the test, which does not necessarily match their level on the target ability.

Importantly, the effective ability that a respondent displays is likely to be influenced by the speed level at which they operated while taking the test. The fact that in most performance settings the expected response accuracy decreases as respondents operate at a higher speed is a common phenomenon in psychology, and is often labeled the “speed-accuracy trade-off.” The speed-accuracy trade-off has been extensively studied in cognitive psychology (Townsend and Ashby, 1983; Luce, 1986) using different experimental methods, such as varying the time when participants are given a signal to respond or varying deadlines for an overview of methods (see e.g., Wickelgren, 1977). While the type of tests used in these studies do differ from standard ability tests, the speed-accuracy trade-off phenomenon likely holds to some degree for most ability tests as well, as one can generally assume that reducing the amount of resources available reduces the expected quality of the outcome of the response process but for some exceptions (see Walczyk et al., 1999; Walczyk and Griffith-Ross, 2006).

Since our focus is on the use of IRT models for the estimation of ability, it may be most helpful to focus on the trade-off as formulated at the level of *effective ability* and *effective speed* (van der Linden, 2009), since in IRT the expected response accuracy is a function of both item and person parameters, and since in IRT the focus is generally on measuring a respondent's (latent) ability rather than (manifest) accuracy. Thus, our focus will be on the *speed-ability trade-off* (abbreviated as “SAbT” and used as shorthand for “effective-ability effective-speed trade-off”) rather than the speed-accuracy trade-off (for which the abbreviation “SAT” is commonly used). Consequently, it makes sense to for each person see effective ability as a function of effective speed (van der Linden, 2009), and in line with the literature we will refer to this person-specific function as the SAbT function (Goldhammer, 2015). This SAbT function informs us what a respondent's level of effective ability is for any given level of effective speed.

Here, it may be useful to consider the hierarchical modeling framework for response time and accuracy, which jointly models effective speed and effective ability (van der Linden, 2007). In this framework, effective speed^2^ (denoted by τ) is taken to be a latent variable that explains response time, and effective ability (denoted by θ) is taken to be a latent variable explaining response accuracy. Commonly, a log-normal model is proposed for the response time *T* of respondent *p* on item *i* (van der Linden, 2006):

(1) $$\begin{array}{l} T_{pi}\sim logNormal\left(\xi_{i}-\tau_{p},\sigma_{i}^{2}\right), \end{array}$$

where ξ_*i*_ is a time-intensity parameter of the item that captures how much time on average respondents spend on the item, and where $\sigma_{i}^{2}$ is the residual variance in log-response time. For modeling response accuracy, standard IRT models are commonly used to capture the relationship between θ and the expected item scores.

The hierarchical modeling framework commonly assumes that a respondent's effective speed and effective ability are constant throughout the test (van der Linden, 2009). At the population level, τ and θ can be correlated, accounting for the possibility that respondents who choose to work fast may also be highly able. It is important to note however that this correlation concerns the *between-person* association between effective speed and effective ability, which may be very different from the *within-person* association between effective speed and effective ability (i.e., the SAbT), which cannot be modeled based on test administrations where speed is taken to be stable throughout the test (van der Linden, 2009). Thus, it may be that more capable test-takers also choose to work at a higher speed than average, resulting in a positive correlation between speed and ability (see e.g., van der Linden et al., 2007; Bolsinova et al., 2017), even though the within-person association between effective speed and effective ability can generally be assumed to be negative in ability testing (van der Linden, 2009).

Methods for modeling the SAbT function based on data collected under varying time constraints have been proposed (Goldhammer et al., 2017)^3^. These methods allow for estimating a general SAbT function (which captures the rate at which effective speed on average is traded for effective ability) as well as person-specific deviations from this average trade-off function (e.g., with some respondents sacrificing more effective ability than others when increasing speed). However, for these methods to work, for each respondent observations of their performance at different levels of speed are needed (Goldhammer et al., 2017), which are unavailable in standard testing applications.

Unfortunately, if respondents differ in their effective speed the measurement of ability will always be confounded by a respondent's choice with respect to their chosen speed-ability balance (van der Linden, 2009). That is, by working at different levels of speed, respondents are effectively taking the test under different conditions, and the comparability of their performance is confounded by these differences. Without information about each respondent's SAbT function, this means that performances of respondents operating at a different speed are in principle not comparable. Thus, only if all respondents would operate at the exact same speed would their performance be directly comparable (Goldhammer, 2015).

**Figure 1:**
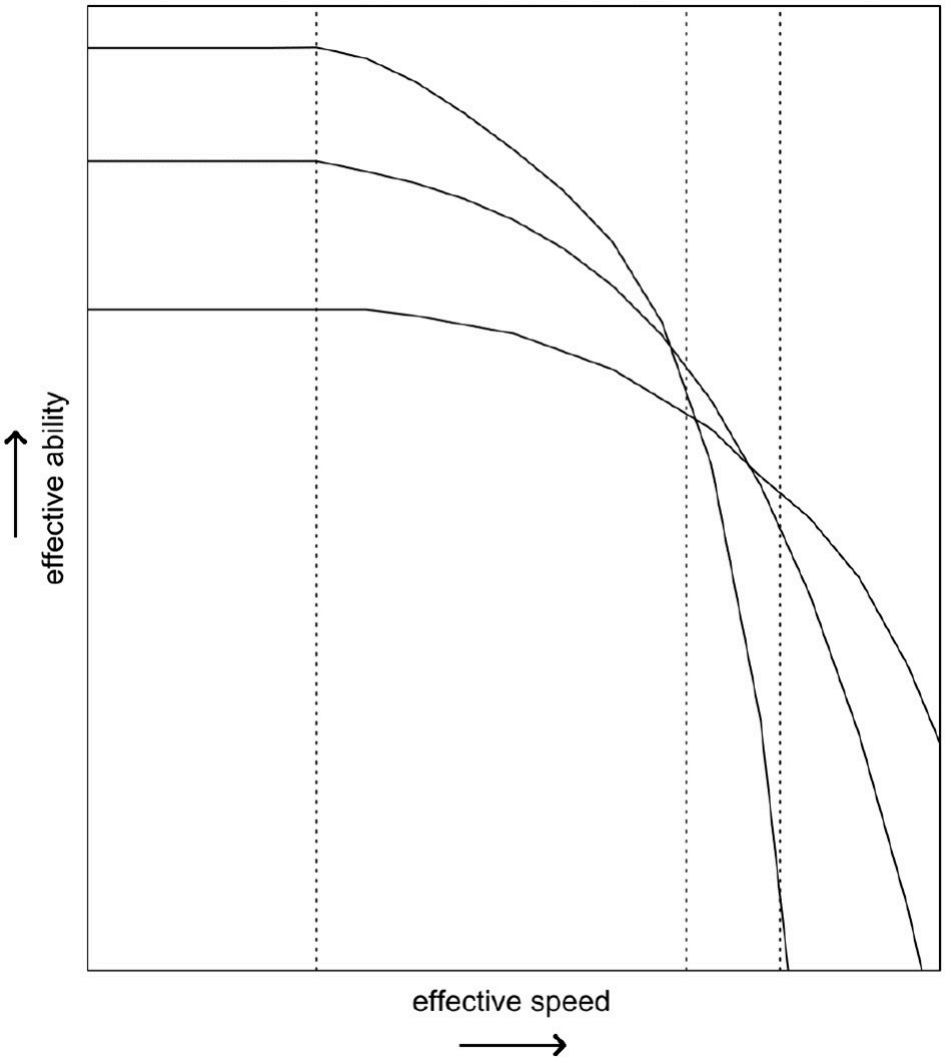
The hypothetical speed-ability trade-off functions of three respondents. The three vertical lines represent different testing conditions, where different minimal levels of effective speed are imposed.

The SAbT informs us that what the test measures is always *effective ability given a certain speed*. As respondents may differ in their SAbT function, there does not need to be a one-to-one correspondence—both in terms of the absolute level and in terms of the ordering of respondents on that ability—between the effective ability obtained at one level of speed and the effective ability obtained at another level of speed (Bolsinova and Tijmstra, 2015). This is illustrated in Figure 1, where it can be observed that depending on which level of speed is considered, three fictional respondents change in their ordering on the measured ability. This example shows that it would at the very least be incomplete to talk about “the ability as measured by the test” without making explicit reference to the speed at which the test was supposed to have been taken, because as this speed increases the measured ability may change both quantitatively (i.e., lower values of θ) and qualitatively (i.e., different person orderings, reflecting a change in the exact attribute being measured).

### 2.2. Operationalization of ability in maximum performance tests with time limits

IRT models for maximum performance tests commonly ignore the item response times and only use the response accuracy for the estimation of ability. This means that what is estimated is the *effective ability* of the respondents, ignoring possible differences between respondents in their effective speed. This may be defensible in pure power tests (Thurstone, 1937; Gulliksen, 1950), where maximum performance without any time limit is sought after: It is up to respondents to comply with the test instruction to show maximum performance, and hence they should fully favor accuracy over speed. Hence, the target ability on pure power tests can be operationalized as effective ability obtained when operating at infinitely low speed, in line with Thurstone's (1937) definition: “[t]he ability of an individual subject to perform a specified kind of task is the difficulty E at which the probability is 1/2 that he will do the task in infinite time.”

On power tests the SAbT function can be expected to level off as speed decreases^4^, meaning that there is a maximum (non-infinite) level of effective ability that the respondent is able to display that can be realized without assuming the respondent to work at infinitely low speed. Once the SAbT function levels off, working at a lower speed does not notably improve performance. Somewhat more leniently one could thus state that on non-speeded tests the target ability is operationalized as the effective ability obtained when operating at that speed level where further slowing down will not increase the effective ability.

Both operationalizations result in pure power tests being intended to measure a respondent's maximum realizable level of effective ability. To the extent that respondents operate at a higher level of speed than intended by the test (i.e., to some extent sacrificing effective ability for speed), such respondents can be considered to show a form of non-compliance, in the sense that they do not take as much time as they need to show maximum performance. This non-compliance will result in a confounding of measurement of ability in the same way that for example low motivation would. Under the assumption that respondents have complied with the test instructions, a comparison of their displayed performance and estimated ability level based on a pure power test can be considered fair.

In contrast with such pure power tests, in this paper we will refer to power tests with an effective time limit—that is, tests where some respondents have to sacrifice some effective ability to manage completing all the items within the time limit—as *speeded power tests*. We prefer this terminology over using the term “partially speeded test” (Lord and Novick, 2008, p. 132), because “speeded power test” makes it clear that what is at stake is still only the assessment of “power,” but that this is done under time constraints (i.e., forcing a minimal level of speed on the respondent). This definition implies that any test where the time constraint forces some respondents to showcase a lower level of effective ability than they could display with infinite time (i.e., most applications of maximum performance tests) constitutes a speeded power test, which is in line with the idea that pure power tests do not exist in practice (van der Linden and Hambleton, 1997, p. 166).

The operationalizations of ability as proposed for pure power tests may be problematic for speeded power tests, as it directly conflicts with the test instruction to complete the items within a certain finite time period and hence to sacrifice effective ability if necessary to achieve this. Here, two ways of approaching this imposed time limit can be considered: treating the time restriction as a nuisance factor disturbing the measurement of the intended ability, or treating the time restriction as playing a role in the operationalization of ability. While the first approach is often implicitly embraced in practice, where the speededness of the test is commonly ignored, this does entail a fundamental mismatch between the ability that is supposed to be measured and the tools that are used to measure it. That is, if the aim is to measure someone's maximum realizable effective ability, then to the extent that the time limit forces some respondents to operate at a faster speed than is needed realize this maximum effective ability, this test does not measure the intended ability. It may also be noted that this confounding is due to compliance rather than non-compliance with the test instructions, meaning that only for respondents who adopt an adequate speed rather than take as much time as needed measurement is confounded.

In applications where not reached items are prominent it is difficult to defend that respondents do not have to sacrifice effective ability to be able to complete the test in time, since this is likely exactly why not everybody succeeds in completing the test. That is, having items that are not reached by a notable proportion of respondents is a clear indication that the test is speeded. Problematically, the extent to which measurement of the maximally attainable effective ability is confounded in such speeded settings may be difficult to determine, because even if most respondents do reach the end of the test, they may have had to work at a faster speed than what allows them to reach their maximum effective ability.

A less problematic approach may be to treat the speededness of the test as a fundamental part of the test and of the operationalization of the measured ability. Just as the target ability in tests without a time limit can be taken to correspond to effective ability at infinitely low speed, one can take the target ability of a speeded power test to be the effective ability at the intended level of speed, for example the level of speed at which they have a 90% chance to complete the test in time^5^. This level of speed that the test expects the respondents to adopt can be referred to as the *target speed* of the test, and the effective ability at the target speed constitutes the *target ability*.

By incorporating the speededness of the test into the operationalization of ability, the test and the target ability are in alignment. If it is clear to respondents that they should aim at showing as good a performance as possible on the test, the fact that effective ability decreases as speed increases can be taken to imply that they should adopt a speed that allows them to just be able to complete the test. This means that they comply with two important parts of the instructions: (1) Do your best to complete all items, and (2) try to perform to the best of your ability on these items. If they would speed up further, accuracy would suffer further and their performance would be lower than it would be if they had worked at the minimum allowable speed level, while if they slowed down more they would not be able to complete all the items. That is, choices of speed deviating in either direction from the target speed should be considered problematic for the respondent who wants to comply with the test instructions^6^.

We argue that the target ability of a test is not necessarily the effective ability given which the responses came to be (i.e., the effective ability matching the actual effective speed), but rather the effective ability as would be obtained when operating at the target speed of the test. Let us by τ^*^ denote the target speed, by θ^*^ denote the target ability, and by τ and θ the actual effective speed and effective ability. For dichotomous item scores we can then extend the standard two-parameter logistic model (Birnbaum, 1968) capturing the probability of a correct response of a respondent *p* to an item *i* as follows:

(2) $$\begin{array}{l} \mathrm{Pr}\left(X_{pi}|\theta_{p}^{*},\tau_{p}\right)=\frac{1}{1+\exp\left(-\alpha_{i}\left(\theta_{p}^{*}+h_{p}\left(\tau^{*}-\tau_{p}\right)-\beta_{i}\right)\right)}, \end{array}$$

where $h_{p}\left(\tau^{*}-\tau_{p}\right)$ denotes a possibly person-specific monotonic non-decreasing function relating a decrease in effective speed to an increase of effective ability, and where α_*i*_ and β_*i*_ denote the item discrimination and difficulty parameter, respectively. We can consider

(3) $$\begin{array}{l} g_{p}\left(\tau_{p}\right)=\theta_{p}^{*}+h_{p}\left(\tau^{*}-\tau_{p}\right). \end{array}$$

to represent the SAbT function for respondent *p* on the test that is considered. As discussed in the previous subsection, this person-specific SAbT function cannot be estimated from a single test administration in which a respondent is assumed to work at a constant speed, but rather requires observations of a respondent's performance while operating at different levels of speed (Goldhammer, 2015).

### 2.3. Illustrative example

**Figure 2:**
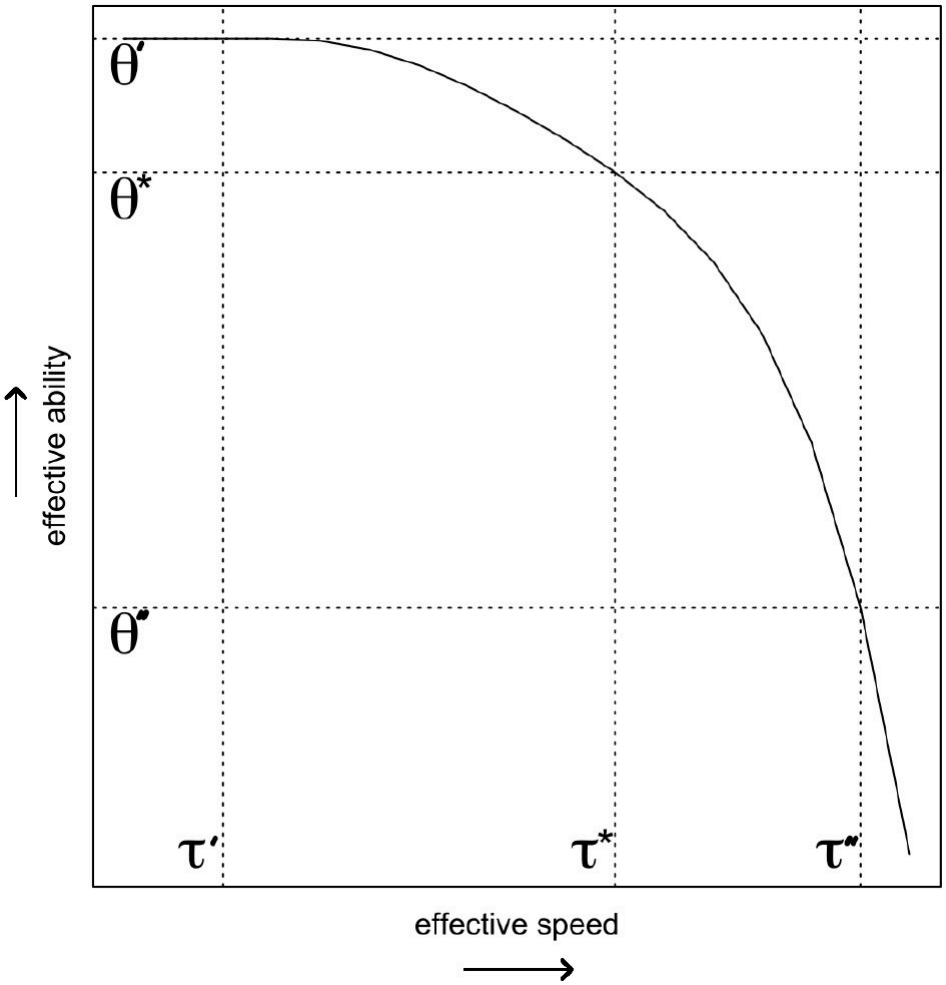
The speed-ability trade-off function that is shared by the three hypothetical respondents. The vertical lines indicate the different levels of effective speed discussed in the example, and the horizontal lines capture the level of effective ability of these persons at those levels of effective speed (i.e., their level on θ′, θ*, and θ″).

Adopting the idea that the speededness of a test should be part of the operationalization of the target ability has important consequences for how to deal with not reached items, as can be illustrated using a hypothetical case study. Imagine respondents A, B, and C, who take an ability test with open-ended questions that consists of 20 equally difficult and time-intense items, and which imposes a test time limit. These three respondents happen to have exactly the same SAbT function (see Figure 2), and hence whenever they operate at the same level of speed they will all share the same level of effective ability^7^.

Imagine that the three respondents differ notably in how they approach the test's time restriction. Respondent A takes careful stock of the amount of available time, adjusts her speed to the intended target level speed τ^*^ at the start of the test, and completes all 20 items. Both respondent B and C do not pay heed to the test time limit, and start working at a slower and for them more comfortable speed level τ′ for the first 9 items. At that point, 90% of the available time has passed, and they realize that at this rate they will never complete all the items in the test. In line with the test instructions, respondent B aims at completing all the items in the test, forces herself to speed up tremendously to achieve this (τ″), and completes all 20 items. Respondent C decides to stick to her more comfortable speed level τ′, completes one more item before the time runs out, and does not reach the last 10 items on the test.

While the three respondents share the same SAbT function, the effective ability that they display on the test differs markedly, as illustrated in Figure 2. Respondent A worked at the target speed, and hence her effective ability $\theta_{A}=\theta^{*}$. Both respondent B and C start out operating at ability level θ′ matching their speed τ′, and hence have a higher expected accuracy than respondent A on the first nine items. Finally, respondent B operates at θ″≪θ′ when she decides to speed up to complete the test in time. Hence, on the last 11 items respondent B displays an effective ability that is much lower than the effective ability with which she answered the first 9 items, and that is also lower than θ_*A*_.

Since the level on the target ability is the same for all three respondents, any structural differences between the performances of these respondents can be thought to constitute a form of differential item functioning (DIF; e.g., see Mellenbergh, 1989), in the sense that person differences other than the ability of interest are needed to explain the observed responses. If this DIF is not corrected for, the estimated ability as obtained based on the IRT model will be biased, leading to an unfair comparison of persons. This makes it clear that differences in operating speed may confound measurement, just like differences in motivation might.

One would ideally like to recover the same level on the target ability for all three respondents, despite their differences in test taking behavior. While deviations from the test instructions such as displayed by respondent B and C should ideally be prevented as much as possible, it is unlikely that this can fully be achieved in practice. Thus, the question to what extent the different methods for dealing with not reached items enable us to correctly recover the target ability remains of practical importance.

### 2.4. Applying the three approaches to the example

The expected ability estimate of respondent C who did not reach 10 out of 20 items due to inadequate speed depends on how not reached items are treated. In the first approach the fact that the last 10 items were not reached is treated as ignorable and will not be taken into account in estimating her ability. Thus, for this approach, the estimate ${\hat{\theta }}_{C}^{*}$ is expected to be close to $\theta_{C}^{\prime }$ and hence to be larger than $\theta_{C}^{*}$, since on the completed items she was able to work using a higher level of effective ability than if she had worked at the target speed.

In this example the missingness is not completely at random (Rubin, 1976), but depends directly on a respondent's chosen level of speed. This would not be problematic if the speed at which someone worked is independent of the level of effective ability that they display while working at that speed, in which case the missingness can be considered ignorable. However, as the discussion of the SAbT makes clear, it is generally highly implausible that the speed at which someone operates on a test where finishing on time is demanding is independent of the ability that the respondent displays while working at that speed.

By ignoring the fact that respondent C did not comply with the instruction to complete the test in time, the procedure ignores the fact that she did not sacrifice enough effective ability to reach the intended target speed, and effectively pretends that she could have answered the last 10 items while operating at θ′ but simply did not do this. That is, by treating the missingness as ignorable, the procedure assumes that the responses *could* have been observed but simply were not, and also that *if* these responses would have been observed, they would have been in line with the performance on the first 10 items. But this counterfactual scenario is simply not possible, since there is not enough time available for this. Thus, in the presence of a SAbT this procedure structurally overestimates the target ability of those who did not adopt an adequate speed level to complete the test in time.

The contrast between what happens to the estimated ability of respondent B and C when using this approach is especially striking. They behaved in the same way for most of the test, working at an inadequate speed. Respondent B, presumably assuming that it is better to attempt to quickly answer 11 items rather than leaving 10 unanswered, adjusted her speed accordingly. However, this results in 11 of the 20 items having been dealt with at θ″, leading to a notable performance drop compared to the first 9 items. Overall, her estimated ability will be much lower than the estimate for respondent C based only on her observed responses^8^. Thus, while respondent B increased her speed to complete all the items and respondent C did not, the procedure “rewards” respondent C with an overestimate of her ability while “punishing” respondent B with a much lower overall estimated ability for following the instructions and completing the test in time.

The issue of “rewarding” working at an inadequate speed seems intuitively to be avoided by the 0-scores imputation approach. With this imputation, the difference in performance between respondent B and C is likely to be much smaller, with respondent C failing all the last 10 items on the test, and respondent B likely failing most of the last 11 items due to working at a very low θ″. Thus, for both of them their performance on the second part of the test reflects an effective ability level that is much lower than θ^*^. However, this will partly be compensated by their improved performance on the first half of the test. Thus, for respondent *B* the overall estimated ability is expected to lie between θ′ and θ″, and for respondent *C* somewhere between θ′ and −∞ (i.e., the effective ability displayed on the items with imputed 0-scores).

Whether after imputing 0-scores for not reached items the estimated ability of respondent C will be close to θ^*^ will depend on the shape of the SAbT function, the differences between the effective speed and the target speed, and the proportion of not reached items and properties of these items. However, one can at least conclude that this approach treats the performance of respondent B and C as much more comparable than the approach where the missingness is ignored. Additionally, it is clear that this imputation approach corrects the ability estimate of respondents with not reached items in the right direction toward θ^*^. However, it is not clear whether this correction is of the right magnitude: The procedure can over- or undercorrect^9^.

The issues with the first approach seem to arise from treating missingness as ignorable. Prima facie, this gives some appeal to methods that fall under the third approach, which model the missingness mechanism, for example by including the number of not reached items as a predictor of ability (Rose et al., 2010, 2017; Rose, 2013). However, what is needed is information about what *a particular respondent's* effective ability would have been if they had operated at the target speed. Crucially, this is a counterfactual within-person question, which as discussed in section 2.1 cannot be answered by considering the association between effective ability and effective speed or the number of not reached items, which is observed at the between-person level (van der Linden, 2009). The desired information at the within-person level is simply not available in standard testing practice.

In the absence of the information about a respondent's SAbT function, it can be dangerous to substitute the needed person-specific within-person association with the between-person association between effective ability and the proportion of not reached items. For example, if most people taking the test have similar SAbT functions [i.e., have similar *h*_*p*_(τ) and θ^*^], then those respondents that reached all items will on average have displayed a lower level of performance on the completed items than respondents who took more time on each item and as a result did not reach all items. This would result in a positive association between the amount of not reached items and the effective ability. If this mechanism would be incorporated in the estimation of ability, the ability estimates of respondents with not reached items would be corrected in the *wrong* direction (i.e., with ability estimates that are larger still than θ′, let alone θ^*^). Morevoer, even if the between-person and within-person association are in the same direction, this still leaves the question of whether the correction of the ability estimate is of the right magnitude unanswered.

These conclusions show that none of the three approaches are optimally equipped to deal with missingness due to not reached items, and that the estimates of the target ability may be highly biased for some of the procedures. Concretely, one can predict that approach 3 outperforms approach 1 only if respondents who have not reached items on average show a *lower* level of effective ability than respondents who do. Approach 2 can be expected to outperform the other two approaches when working at the target speed means that respondents generally have made a notable sacrifice in effective ability (compared to their maximum level of effective ability), while on tests where this sacrifice is rather small (i.e., it being hardly speeded) it will overcorrect. To study the exact performance of these three different approaches under a range of relevant scenarios, a simulation study was conducted, which is presented in the next section.

## 3. Simulation study

### 3.1. Method

We performed a simulation study to investigate to what extent the estimates of ability obtained when using different ways of handling not reached items match the target ability θ^*^ (i.e., effective ability given the target speed). In this study we defined target speed as the speed at which the probability of completing all the items is 0.9^10^. We considered a test with 40 items with time intensities of all items equal to 2 and all residual variances of log-transformed response time equal to 0.1. The value of the target speed was determined using Monte Carlo simulation. For 501 equidistantly spaced values of τ ranging from −1.5 to −1, 100,000 vectors of response times in seconds were generated under the lognormal model in (1). The value of τ for which the proportion of generated response time vectors with the total time below the time limit of 1,200 s was the closest to 0.9 was chosen as the target speed (τ^*^ = −1.287).

The sample consisted of 5,000 respondents, for which the values on the target ability θ^*^ were specified as follows: For the first 2,500 respondents we used the quantiles of the standard normal distribution, for which the cumulative probabilities were equally spaced between $\frac{1}{2501}$ and $\frac{2500}{2501}$; these values were repeated for the second half of the sample. The same true values of θ^*^ were used in all conditions. The sample was taken to consist of two equally sized groups: compliers (*Z*_*p*_ = 1), who operated at the target speed, and non-compliers (*Z*_*p*_ = 0), who operated at a lower speed level. The relationship between θ^*^ and *Z* was a design factor in the simulation, with θ^*^ and *Z* either being independent or dependent. For the independence condition, we assigned the first half of the sample to the group of compliers and the second half of the sample to the group of non-compliers. For the dependence condition, group membership was taken to depend on θ^*^. Here, *Z* was generated using a logistic regression with a coefficient of 1 for the predictor θ^*^, capturing the idea that respondents who need to work more slowly may also be less able. The 5000 values of *Z* were repeatedly simulated until a sample was obtained where the groups were of equal size. For this sample the average θ^*^ of the compliers was 0.813 higher than that of the non-compliers. The same values of *Z* were used in all conditions in which θ^*^ and *Z* were correlated.

For the 2,500 non-compliers, equally spaced values between τ_*LB*_ and τ_*UB*_ were used for effective speed. The upper bound τ_*UB*_ = −1.353 was chosen using Monte Carlo simulation analogous to the one used for determining the value of τ^*^ as the level of speed for which the probability of completing all the items was 50%. The lower bound τ_*LB*_ was varied and functioned as a design parameter. Two different values of τ_*LB*_ were used, corresponding to different expected percentages of missing responses in the group of non-compliers: −1.781 and −1.534^11^, resulting in respectively 20 and 10% of missing responses for the group of non-compliers (about 10 or 5% overall missingness in the sample). The values between τ_*LB*_ and τ_*UB*_ were randomly matched to the respondents in the non-compliers group in the independence conditions. In the dependence conditions, τ was correlated with θ^*^: Respondents with lower ability deviated more from τ^*^ than respondents with higher ability. Here, the values of effective speed were matched to the respondents with different θ^*^ in such a way that the Spearman correlation in the sample between θ^*^ and τ was equal to 0.5.

To capture the impact that working at a lower speed has on the effective ability, SAbT functions needed to be specified, as can be observed in Equation (2). For the sake of simplicity, in this simulation we used a person non-specific linear function for the SAbT: $h_{p}\left(\tau^{*}-\tau_{p}\right)=\gamma \times \left(\tau^{*}-\tau_{p}\right)$ for all respondents. The larger the positive SAbT parameter γ, the larger the increase in effective ability that is achieved when decreasing the effective speed. Empirical research by Goldhammer et al. (2017) on the relationship between effective speed and ability in tests with time limits suggested that γ = 3.3 may be realistic for tests consisting of simple cognitive tasks with strict time limits, which we adopted for the “strong SAbT” condition. Additionally, we considered a “weak SAbT” condition where the test was taken to be less speeded, for which we used γ = 3.3/2 = 1.65. In this “weak SAbT” condition the speed-ability trade-off is half as strong as found in the study of Goldhammer et al., which may be more in line with standard ability testing settings^12^.

Thus, three design factors with two levels each were considered, resulting in eight simulation conditions. Table 1 summarises the different conditions. It contains the correlation between the effective speed and effective ability (which is not the same as the correlation between effective speed and the target ability) and the average difference between the effective ability and the target ability in the group of non-compliers. In each condition 1,000 sets of data were generated. First, the response times in seconds were generated under the log-normal model. Second, for those items for which the cumulative response time was smaller than 1,200 s, the response accuracy was generated under the 2PL with effective ability as the person parameter, while items for which the cumulative response time exceeded 1,200 resulted in missing values. The discrimination parameters of all the items were equal to 1; the difficulty parameters of the items from 1 to 10, from 11 to 20, from 21 to 30, and from 31 to 40 were set to [1, −1, 0, −0.5, 0.5, −1, 1, 0, 0.5, −0.5].

**Table 1:** Overview of the properties of the 8 conditions used in the simulation study.

**PM (%)**	θ^*^ and *Z* related?	**SAbT**	***Cor*(τ, θ)**	$E\left(\theta^{*}|\;Z=0\right)$	**E(θ| Z=0)**
10	No	Strong	−0.47	0	0.92
10	No	Weak	−0.27	0	0.46
10	Yes	Strong	−0.03	−0.41	0.52
10	Yes	Weak	0.28	−0.41	0.06
5	No	Strong	−0.27	0	0.52
5	No	Weak	−0.14	0	0.26
5	Yes	Strong	0.32	−0.41	−0.01
5	Yes	Weak	0.43	−0.41	−0.20

The first three columns refer to the design factors. PM refers to the overall percentage of missing responses, SAbT refers to the strength of the speed-ability trade-off, θ^*^ and θ refer to the target ability and effective ability, respectively, and Z refers to group membership (Z = 1 for compliers and 0 for non-compliers).

The estimates of θ^*^ were obtained with three different methods, in line with the three different approaches discussed in the Introduction. The first method (labeled “Ignorable”) assumes the missingness to be ignorable. For this method the item parameters of the 2PL were estimated using marginal maximum likelihood given the observed data only, and the EAP estimates of ability were obtained. The method in line with the third approach (labeled “Latent Regression”) treats the missingness as non-ignorable and makes use of a latent regression model. Here, the possible non-ignorability of the missing data was modelled by including the number of completed responses as a linear covariate for ability (e.g., see Rose et al., 2010). Finally, the method labeled “Imputation” makes use of imputed 0-scores for not reached items. Here, the item parameters of the model were estimated while treating the missing values as ignorable, while the expected a posteriori (EAP) estimates of ability were obtained given the response patterns in which zeros were imputed for the missing values^13^. In all cases the R-software (R Core Team, 2017) and the R-package mirt (Chalmers, 2012) were used, and the models were estimated using an EM-algorithm with numerical integration with 61 quadrature points. In all cases the IRT scale was identified such that the average value of ability in the full sample was equal to zero.

In all conditions, average estimates of the respondents' ability across 1,000 replications were computed for the three estimation methods. The estimates were compared with the values of θ^*^ used to generate the data. It may be noted that we are comparing the estimates not with the effective ability with which the respondents performed (as has been the focus of the simulation studies mentioned in the Introduction) but with the *target ability* of the test; the effective ability which the respondents would have had if they had performed at the target speed. The methods were compared based on the following outcome variables: (1) average bias of the estimated ability in the group of non-compliers, (2) average bias of ability in the group of compliers, (3) average absolute bias in the group of non-compliers, (4) average absolute bias in the group of compliers, (5) the correlation between θ^*^ and the estimates (averaged across replications). Absolute bias was considered in addition to bias because it provides additional information in situations when bias is positive for some respondents and negative for others.

### 3.2. Results

The results of the simulation study are presented in Table 2. The results of Method Ignorable show that treating the not reached items as missing values that can be ignored results in a structural bias of the person parameters, showing positive bias for the respondents in the group of non-compliers (*Z* = 0), and (because the procedure assumes θ^*^ to have a standard normal distribution in the population) showing negative bias in the group of compliers. This bias is strongest in the conditions where the effect of slowing down is larger (i.e., strong SAbT) and in the condition with a relatively large (i.e., 10%) proportion of not reached items. These are also the conditions in which the absolute bias is largest and the correlation between the estimated ability and the target ability is lowest.

**Table 2:** Results of the simulation study, based on 1000 replications.

**Method**	**PM**	θ^*^ and *Z* related?	**SAbT**	**Bias**		**Absolute bias**		$Cor\left(\hat{\theta },\theta *\right)$
				***Z* = 0**	***Z* = 1**	**Z = 0**	**Z = 1**	
Ignorable	10	No	Strong	0.36	−0.36	0.44	0.37	0.83
			Weak	0.19	−0.19	0.25	0.21	0.91
		Yes	Strong	0.47	−0.47	0.51	0.47	0.76
			Weak	0.24	−0.24	0.27	0.24	0.89
	5	No	Strong	0.22	−0.22	0.26	0.23	0.90
			Weak	0.11	−0.11	0.16	0.14	0.93
		Yes	Strong	0.27	−0.27	0.27	0.27	0.89
			Weak	0.15	−0.15	0.16	0.15	0.92
Latent	10	No	Strong	0.45	−0.45	0.52	0.45	0.80
Regression			Weak	0.23	−0.23	0.28	0.24	0.90
		Yes	Strong	0.47	−0.47	0.51	0.47	0.76
			Weak	0.20	−0.20	0.22	0.20	0.90
	5	No	Strong	0.25	−0.25	0.28	0.25	0.90
			Weak	0.12	−0.12	0.16	0.14	0.93
		Yes	Strong	0.24	−0.24	0.24	0.24	0.90
			Weak	0.10	−0.10	0.10	0.10	0.93
Imputation	10	No	Strong	0.07	−0.07	0.38	0.15	0.89
			Weak	−0.06	0.06	0.28	0.11	0.91
		yes	strong	0.18	−0.18	0.24	0.19	0.90
			Weak	0.02	−0.02	0.13	0.03	0.93
	5	No	Strong	0.09	−0.09	0.24	0.13	0.92
			Weak	−0.01	0.01	0.18	0.08	0.93
		Yes	Strong	0.16	−0.16	0.18	0.16	0.92
			Weak	0.06	−0.06	0.10	0.06	0.94

Bias and absolute bias refer to the average (absolute) bias of the estimate of the target ability in the groups compliers (Z = 1) and non-compliers (Z = 0). PM stands for the percentage of missing responses in the full sample, SAbT refers to the strength of the speed-ability trade-off, θ* refers to the target ability, $\hat{\theta }$ refers to the ability estimate produced by the method, and $Cor\left(\hat{\theta },\theta *\right)$ is the correlation between the estimates and the true values of target ability (averaged across replications).

Compared to the results of Method Ignorable, Method Latent Regression performs worse in the conditions where the correlation between τ and θ is negative^14^, and performs better in the three conditions where that correlation is positive (see Table 1). In those three conditions, bias and absolute bias are smaller and the correlation between $\hat{\theta }$ and θ^*^ is larger than when Method Ignorable is used, but the converse holds when there is a notable negative relationship between τ and θ, meaning that which of the two methods performs better is fully determined by the sign of the between-person association between these two variables.

Compared to the other two methods, Method Imputation showed better performance in terms of bias and absolute bias in all eight considered conditions, and in most conditions also showed a higher correlation between the estimated ability and the true value for the target ability. Similar to the other procedures, the bias and absolute bias were smallest for the conditions where the SAbT is weak. When the SAbT is strong, there is still positive bias for the ability estimates in the group of non-compliers, indicating that the downwards adjustment of the ability estimate obtained by using imputed zero scores for missing values resulted in an undercorrection in that condition. Thus, even though using Method Imputation notably reduced the bias compared to the other two methods, in the presence of a strong SAbT a correction even stronger than the one imposed by imputing zero scores would have been necessary to fully eliminate the bias in the ability estimates. Unlike the other methods, the proportion of not reached items did not notably influence the overall bias, although the absolute bias was higher when the proportion of missings was higher. When θ^*^ and *Z* are related, the method results in estimates of θ^*^ for the non-compliers that are higher than when θ^*^ and *Z* are not related, as is reflected in the average bias in these conditions. For the group of non-compliers, the average absolute bias was lower when θ^*^ and *Z* were correlated than when they were not. This effect may be explained by considering the fact that in the conditions where θ^*^ and *Z* are related the average θ^*^ in the group of non-compliers is lower than in the conditions where θ^*^ and *Z* are independent. That is, imputing 0-scores has a stronger impact on the estimated ability of respondents with a relatively high effective ability, compared to respondents with a relatively low effective ability.

**Figure 3:**
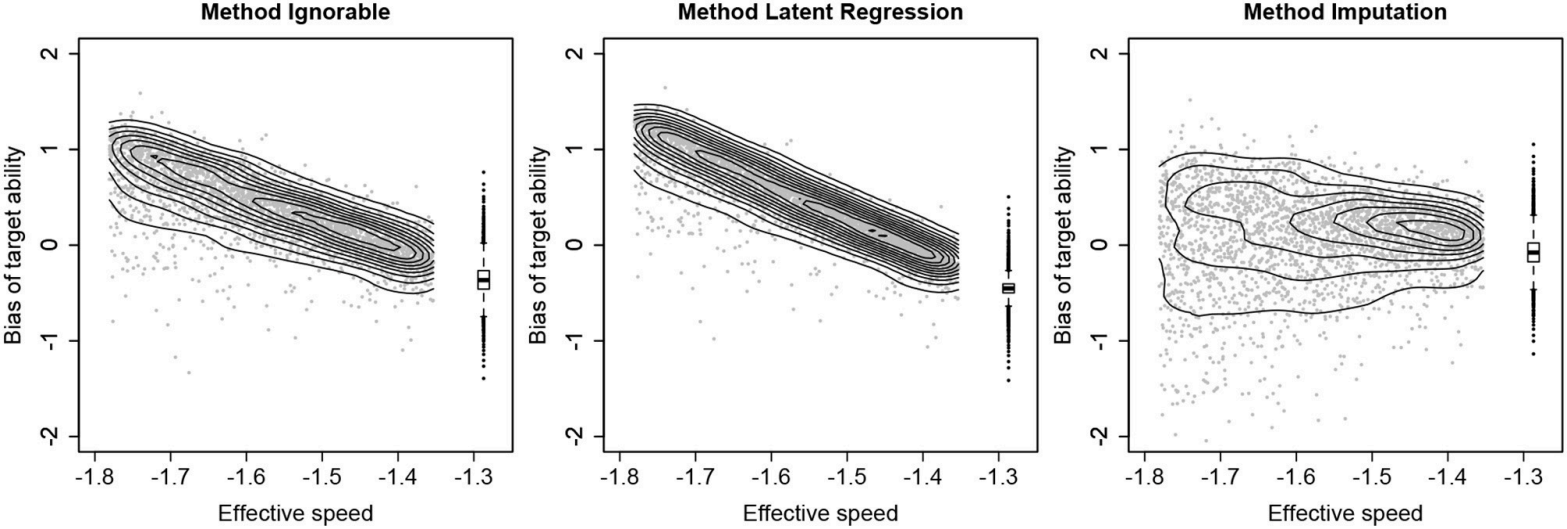
Relationship between the effective speed (on the *x*-axis) and the bias of the target ability (on the *y*-axis) for the condition with 10% missing responses in the full sample, a strong speed-ability trade-off and the target speed and group membership (*Z*) being independent. The results for non-compliers are shown in a scatterplot with density contours, and the results for compliers are shown in a boxplot.

To further illustrate the differences between the three methods, Figure 3 shows how the bias of target ability varies across persons and how it depends on the values on the target speed. The results are visualized for the first condition from Table 1 (i.e., 10% missing responses in the full sample, strong SAbT, and unrelated θ^*^ and *Z*). Here it can be seen that for both Method Ignorable and Method Latent Regression, there is a strong relationship between the deviation from the target speed and the size of the bias, a pattern that appears to hardly be present for Method Imputation. It may be noted that for each method for a given speed level there still is variation in the bias, which is due to the fact that ability estimates are shrunk to the mean and persons differ in the size of this shrinkage effect.

## 4. Discussion

Existing approaches that treat not reached items as missing rather than incorrect responses generally focus on obtaining an unbiased estimate of the ability as it is displayed on the reached items. This is either done by treating the not reached items as not administered, or by using information about the missingness as a predictor of the ability to be estimated. In both cases, this effectively entails treating the speededness of the test as a nuisance that may cause some respondents not to complete the test, but the measured ability is still taken to be identical to the ability that would have been measured if the test were untimed. This may make sense in tests where the time limit is hardly effective, and where the minimum speed level that people are forced to work on hardly results in a reduction in effective ability compared to their effective ability in an untimed administration of the test. However, to the extent that this holds, these power tests can practically be considered not to be speeded, and respondents should not have had issues reaching the end of the test within the time limit.

As soon as one considers speeded power tests (i.e., where respondents are pushed to work at a speed level that forces some of them to sacrifice some effective ability), having not reached items can be taken to imply that for those respondents the *level of effective ability displayed on the items that were reached* is higher than the *target ability that the test intends to capture*. Hence, for these respondents the intended inferences are not about their level of effective ability as they displayed on the items that they completed, but rather about the counterfactual level of effective ability that they would have displayed had they worked at the appropriate level of speed.

For speeded power tests there will be some degree of confounding if for respondents with not reached items the ability as displayed on the reached items is taken to be the ability of interest. The degree to which this is problematic will depend on the proportion of respondents that have not reached items, the proportion of not reached items, and the extent to which the target speed level results in a reduced effective ability compared to conditions where more time would be available. This was illustrated in the simulation study, where both the approach that treats the missingness as ignorable and the approach that includes the number of not reached items as a covariate in the measurement model resulted in a consistent overestimation of the target ability for respondents who did not reach all the items on the test when a speed-ability trade-off is present.

As the simulation study shows, using the number of not reached items as a covariate in the model does not structurally improve the estimates of the target ability for respondents with not reached items compared to treating the missingness on these items to be ignorable. Rather, this approach only improves these estimates when the between-person association between effective speed and effective ability is positive, which may not always hold in practice. If this between-person association is indeed positive, the ability estimates of respondents with not reached items are adjusted downwards, but whether this correction is of the right size depends on the strength of this association as well as the steepness of each respondent's SAbT function. In the conditions that were considered in the simulation study, this correction was always found to be too small, even when the SAbT was weak and the association between effective speed and effective ability was relatively strong, meaning that this method consistently overestimated the level on the target ability of respondents with not reached items. These results indicate that it may be difficult to defend that using the number of not reached items as a covariate will result in unbiased or approximately unbiased estimates of the target ability in situation where respondents can be expected to have sacrificed some effective ability to be able to complete the test in time.

It may seem that the conclusion that methods that treat not reached items as missing run into fundamental problems hinges on accepting that the target ability in speeded power tests is not the same as the target ability on that test if it would not have been speeded (i.e., if the target ability would have been the maximum realizable effective ability rather than the effective ability realized when working at the target speed). However, if we would accept that in speeded power tests the target ability is this maximum realizable effective ability, two important issues arise: The test as it is used is no longer equipped to adequately measure the target ability (as the effective time limit prevents some respondents from displaying this maximum realizable ability), and non-compliance with the test instructions is rewarded if these methods are used. That is, if respondents have to sacrifice effective ability to complete the test in time, then not complying with the test instructions by working at a speed that will not allow you to complete all the items in time will result in higher ability estimates, as the hypothetical example illustrated. This could be especially problematic for analyses based on large-scale international surveys, since it may be plausible that respondents of different countries differ notably in their level of compliance with regard to adopting an adequate level of speed, confounding between-country comparisons.

Treating not reached items as incorrect rather than as having missing values results in a downwards adjustment of estimated ability for respondents with not reached items, compared to what their estimate would be if only the reached items would have been considered for the estimation of ability. The simulation study showed that in the conditions considered, this approach resulted in less bias in the estimates of the target ability. However, as the size of this downwards correction does not depend on any information about each respondent's actual SAbT function, the procedure may over- or undercorrect (as Figure 3 shows), depending on factors that one may argue should not affect the size of this correction. For example, the penalty will be more severe for a respondent with a high target ability than for a respondent with a low target ability, as a sequence of 0-scores has more impact for a respondent for whom we would expect many 1-scores than for a respondent for whom 0-scores on these items would have been relatively plausible if they had answered the items. Additionally, the penalty will depend on characteristics of the items that were not reached, meaning that if the not reached items were easy items, the penalty will be more severe than if the not reached items would have been difficult items, again because for the latter items a pattern of 0-scores would be more plausible than for the former. Thus, while imputing 0-scores will adjust the estimated ability in the right direction (assuming a speed-ability trade-off to be present), the size of this correction may not be adequate and hence this method also does not present an optimal general solution for dealing with not reached items.

With all three types of statistical approaches to dealing with not reached items resulting in problems for the estimation of the target ability, the unfortunate conclusion seems to be that current methods do not seem to be equipped to adequately deal with the presence of not reached items for the estimation of ability. However, at least three possible solutions to the issue of not reached items confounding the measurement of ability can be considered: working with non-speeded power tests, working with item-level time limits, or explicitly working with a scoring rule that adjusts for not reached items.

The option of working with non-speeded power tests may seem practically infeasible if we follow the idea of Thurstone (1937) that such tests consider ability when infinite time is taken to solve the item. However, if it is plausible to assume that SAbT functions reach an asymptote as speed decreases, this maximum effective ability may actually be attainable in conditions that do not give respondents infinite time to complete the test; it may be sufficient to ensure that no respondent has to sacrifice any notable amount of effective ability to complete the test within the given time limit. However, it is not defensible that the test is a pure power test if there is a non-neglible proportion of respondents with not reached items.

Alternatively, one can avoid the occurrence of not reached items due to working at an inadequate level of speed by working with item-level time limits rather than test-level time limits, as was also proposed by Goldhammer (2015). Such an item-level time limits approach would embrace the speededness of the test, and would be in line with the idea that there is a ‘target speed’ for the test that each respondent should adopt, corresponding to their effective ability. With the advance of computer-based assessment, imposing such (possibly item-specific) item-level time limits in testing practice has become realistic, making it a relevant alternative to working with test-level time limits.

Finally, one can explicitly communicate the use of the 0-imputation method to the respondents, in line with many high-stakes testing applications. In this way, the scoring rule is communicated which makes it clear that it is up to the respondents to *optimize their performance given this scoring rule*. This way, it is up to the respondent to decide whether the gain in effective ability that they get when working at a speed below what is needed to complete all the items is worth the loss of not providing answers to some of the items on the test. It may be noted that this amounts to redefining the target ability from “effective ability when operating at the target speed” to “the level of performance that a respondent is able to display when working under the communicated scoring rule”.

Importantly, these three alternatives not only differ in the testing conditions that are imposed, but also result in different operationalizations of the target ability. While the question which operationalization is closest to the actual ability of interest is highly important, the answer will depend on the particular application that is considered. If what one is interested in is maximum performance without any time constraints, it feels natural to consider the first approach and attempt to realize a test that is as close to a pure power test as possible. If however performance under realistic time constraints is at stake, the other two alternatives may be more appealing. If a comparison of respondents that all operate at the same speed is sought after, imposing item-level time limits may be most optimal, while allowing respondents to optimize their test-taking behavior more flexibly by taking control of their own level of speed may have more ecological validity in other applications.

## Author contributions

JT has led the development of the manuscript, has co-developed its core ideas and the methods of the simulation study, and has been the main writer. MB has co-developed the core ideas and methods of the manuscript, has programmed the simulation study, and has provided feedback on the text of the manuscript.

### Conflict of interest statement

The authors declare that the research was conducted in the absence of any commercial or financial relationships that could be construed as a potential conflict of interest.

## Footnotes

^1^Different ways of handling omitted responses have been studied extensively. Consequences of traditional ways of dealing with missing data (e.g., treating omitted responses as not administered, as incorrect, or as fractionally correct) for estimation of persons and item parameters have been investigated (Lord, 1974, 1983; Ludlow and O'Leary, 1999; De Ayala et al., 2001; Finch, 2008; Rose et al., 2010; Culbertson, 2011) and model-based approaches for dealing with omitted responses occuring not at random have been developed (O'Muircheartaigh and Moustaki, 1999; Moustaki and Knott, 2000; Holman and Glas, 2005; Debeer et al., 2017; Rose et al., 2017).

^2^Here, it is important to contrast *effective speed* (i.e., the speed level at which a respondent operates on the test) with speed (e.g., a capability of the respondent to produce responses with a certain level of accuracy under time pressure). In this manuscript we will always use speed to refer to effective speed, in line with the terminology of the hierarchical modeling framework for speed and ability (van der Linden, 2007).

^3^It may be noted that in the work of Goldhammer et al. (2017) speed is taken to be an observed rather than a latent variable. In the terminology of this paper, their methods therefore consider the time-ability trade-off function rather than the SAbT function, but the two can be assumed to be similar in shape.

^4^It may be noted here that in speed tests a common assumption is that the probability of correctly answering an item goes to 1 as time increases, but that this assumption cannot plausibly be transferred to power tests, where there is a limit to the level of effective ability that can be realized by a respondent.

^5^It may be noted here that since speed is taken to be a latent variable that is stochastically related to the response times, the adopted level of speed influences the observed response times stochastically rather than deterministically, and hence it is necessary to talk about for example the speed at which one has a certain probability to complete the test, rather than the speed at which one is guaranteed to just finish the test in time.

^6^Only if a respondent would be able to work at a higher speed than is necessary without sacrificing effective ability would it not be problematic for them to do this, but that means that their SAbT function has not yet started to show a decrease at that point and hence the test should for them be considered to constitute a pure power rather than a speeded power test.

^7^This does not mean that their item scores will be identical, but rather only that their effective level of ability is the same.

^8^It may also be noted that respondent B would show notable person misfit due to the violation of the stationarity assumption of the model (van der Linden, 2009), since her effective ability showed a large fluctuation.

^9^For example, for respondent C the maximum number correct on the 20-item test is 10, while it is entirely possible that she could be expected to get more than 10 items correct when operating at the target speed. Vice versa, it is also possible that by working slowly, despite only answering 10 items, she was able to answer more items correct in total than she would have done if she had worked at the target speed (e.g., if she is very bad at working at high speed). This all depends on her (unobserved) SAbT function.

^10^As under the hierarchical model RT is stochastically related to speed, one cannot consider a finite value for speed for which one has 100% chance to complete the test in time. We opted for a speed level that ensures 90% probability of completing all items, which captures the aim of the participant to both complete all items (which is likely to happen at that speed level) and to maximize performance (i.e., not sacrificing too much effective ability).

^11^These values of τ_*LB*_ were chosen using Monte Carlo simulation. For 501 equidistant values of τ ranging from −2 to −1.5, the effective speed parameters for 100,000 non-compliers were sampled from $U\left(\tau ,\tau_{UB}\right)$, their response times to the 40 items were generated and the proportion of items which were answered after the time limit of 20 min was computed. The values of τ for which the resulted average number of missing responses was equal to the desired 20 or 10% were selected as τ_*LB*_s.

^12^Without extensive empirical study of the SAbT functions in standard ability testing settings any choice of SAbT function will unfortunately to some degree be arbitrary, but we felt it important to base our parameter values as much as possible on relevant empirical findings.

^13^It may be noted that since the imputed 0-scores due to not reaching the item do not contain any relevant information about the properties of the item, these imputed scores should be excluded from the calibration of the item parameters. If they would be included, they would result in negative bias for the estimated item difficulty parameter.

^14^With the exception of the condition with 10% not reached items, an association between *Z* and θ^*^, and a strong SAbT, where the results for the two methods are comparable. However, in this condition *Cor*(τ, θ) = −0.03, meaning that the negative correlation is practically zero.
